# Hispanic Parents’ Views of Family Physical Activity: Results from a Multisite Focus Group Investigation

**DOI:** 10.3390/children8090740

**Published:** 2021-08-27

**Authors:** Norma Olvera, Amber J. Hammons, Margarita Teran-Garcia, Maria Plaza-Delestre, Barbara Fiese

**Affiliations:** 1 Psychological, Health, and Learning Sciences Department, University of Houston, 3657 Cullen Boulevard Room 491, Houston, TX 77204, USA; 2 Child and Family Science Department, California State University, Fresno, 5300 North Campus Drive, Mailstop FF 12, Fresno, CA 93740, USA; ahammons@csufresno.edu; 3 Integrated Health Disparities Programs, Illinois Extension, Division of Nutritional Sciences, Biomedical and Translational Sciences Department, Carle Illinois College of Medicine, University of Illinois Urbana-Champaign, 904 W. Nevada St., Urbana, IL 61801, USA; teranmd@illinois.edu; 4 Food Science and Technology Program, Agro-Environmental Science Department, Mayaguez Campus, University of Puerto Rico, Call Box 9000, Mayaguez, PR 00681, USA; maria.plaza@upr.edu; 5 Human Development and Family Studies Department, Family Resiliency Center, University of Illinois Urbana-Champaign, 904 W. Nevada St., Urbana, IL 61801, USA; bhfiese@illinois.edu

**Keywords:** physical activity, Hispanic, family, parents, children

## Abstract

Understanding parental views regarding family physical activity is essential to the development of family-focused physical activity interventions. Using a qualitative methodology with thematic analysis and a socio-demographic questionnaire, this study aimed to examine Mexican American and Puerto Rican parental views on child and family physical activity. Sixty-one parents (56 mothers, five fathers) from four sites (California, Illinois, Texas, and Puerto Rico) each participated in a single one-hour focus group session, which included an average of five parents. The findings of this study indicated that parents perceived themselves and their families to be physically active, while some parents believed their children were getting enough physical activity at school and afterschool programs. Walking, bicycling, and playing soccer were the most common physical activities that parents reported engaging in as a family. In addition, some parents shared their preference for exercising without their children. Time constraints along with unsafe neighborhood streets and parks were identified as the major barriers to being physically active as a family. Mothers reported that fathers’ involvement in physical activity and combining a healthy diet with exercise were useful strategies for physical activity promotion. This study provides valuable information regarding Hispanic parental views concerning family physical activity relevant to the design of culturally family-based physical activity interventions for this population.

## 1. Introduction

Obesity is a major health challenge facing children and adolescents in the United States. The obesity rate has tripled among American youth since 1980 [1]. Although obesity affects US children and adolescents of all genders and ethnicities, this condition afflicts a disproportionate number of Hispanic children compared with non-Hispanic white children [2]. Obesity runs in families. Parents with obesity are twice as likely to have an obese child as non-obese parents [3,4]. The link between parent and child obesity status is also well documented among Hispanic families [5,6]. These findings suggest the need to examine parental factors associated with obesity among minority youth, with the overall goal of developing culturally appropriate family-based obesity prevention and treatment interventions.

The supporting evidence indicates that, among other factors, a steady decline in physical activity (PA) contributes to obesity development in youth and adults [7,8]. Parents play a role in influencing their children’s PA during childhood and adolescence [9,10,11]. A systematic review of parental influences on youth PA [12] revealed that children’s (ages 6–11 years) PA level was positively associated with parental support, involvement, and modeling. More recently, Liszewska and colleagues [13] confirmed that parents’ use of positive social control, encouragement to be active, and modeling increased their children’s (ages 6–11) engagement in PA. Consistently, other studies have shown that parental support (e.g., providing financial support, transportation, and encouragement and signing the child up for structured PA) is associated with children’s PA levels [9,14].

Studies have also assessed parental knowledge and attitudes regarding their own and their children’s PA. Parents reported to be aware of PA’s health benefits for themselves and their family members and valued PA [9,13,15]. Furthermore, parents identified strategies to promote PA in their children, such as focusing on activity enjoyment [9]. Research also reveals that parents believed that their children were meeting PA recommendations at school and considered schools to be the main provider of PA for their children [15]. Despite the health benefits of physical activity, parents admitted that engagement in PA was challenging for themselves and their children. These challenges included lack of time, safety concerns, inclement weather, and lack of access to recreation facilities [15].

Limited research has investigated Hispanic parental views of PA regarding their own and their family with children of various ages. Based on existing literature on this area, Hispanic parents perceived themselves to be sufficiently active during the day because of their physically demanding jobs and household-related work [16]. A recent study comparing PA perceptions of Mexican mothers from central Mexico and US Mexican American mothers identified walking as the most common form of PA in both groups of mothers [17]. Regarding their children, Latino mothers of preschoolers perceived PA as beneficial for their child’s brain health and helpful in strengthening children’s bodies to prepare them for future hard labor work [18]. At the same time, Latino mothers considered that the amount of PA should be limited among preschool aged children, and that excessive PA may be harmful to their child’s health (e.g., damaging their internal organs or injuring their body, including broken bones). It is also well-documented that Hispanic families living in under-resourced communities face numerous challenges being physically active. Latino mothers of preschool-aged children identified limited exercise equipment and access to play areas, and overcrowded environments as major barriers for their children to engage in PA [18]. Among Hispanic mothers of school-aged children, studies revealed unsafe neighborhoods, lack of time, and bad weather as obstacles for their children to be active [17,19]. Taken altogether, most of the previous research on Hispanic parental PA views has focused on perceived PA health benefits, adequate PA dosage, and PA barriers for them and their preschool-aged children.

Little is known about the perceptions of Hispanic parents regarding PA attitudes, preferences, behaviors, and socioenvironmental influences on PA from a family perspective. Considering Hispanic parental PA views within a family context is relevant because of their strong endorsement of familial closeness and engaging in activities as a family [20]. Family systems theory (FST) provides a framework to examine how parental factors might influence PA within family members, given that FST focuses on interactions between individuals within the familial context. FST posits that family members are interconnected, where one member’s behavior influences other family members in the system [21]. Family can also be perceived as a system functioning within a more extensive system consistent with the socio-ecological model (SEM). The socio-ecological model (SEM) offers a framework for understanding the intrapersonal and socio-environmental influences on health behaviors [22,23]. Analyzing how these factors interact to influence behavior is critical to the development of interventions promoting PA. Thus, the purpose of this study is to examine, using a social-ecological model, Mexican and Puerto Rican parental views regarding parental factors (e.g., PA engagement and preferences, PA attitudes, perceived challenges, and facilitators) along with social–environmental factors (e.g., spouse influence, places for engaging in PA) that might influence PA within the family.

## 2. Materials and Methods

### 2.1. Research Design

For this study, a qualitative methodology [24] was employed to collect data. A qualitative approach provides a systematic method to explore parental views regarding PA for themselves and their children.

### 2.2. Participants

Sixty-one parents (56 mothers, five fathers) each participated in a single one-hour focus group session, conducted between June 2015 and August 2018. Each focus group conducted during this period included an average of five parents. Participants were recruited from four sites (California, Illinois, Texas, and Puerto Rico). To participate in the study, parents had to be of Mexican or Puerto Rican descent and to have a child between 6–18 years of age. Mexican and Puerto Rican families were selected to participate in this study because they constitute the largest Hispanic subgroups residing in the United States [25].

### 2.3. Recruitment

Recruitment efforts included posting study fliers and distributing them at churches, grocery stores, flea markets, community centers, laundromats, public schools, and recreation centers. Research assistants also contacted community organizations that serve Hispanic families to ask for referrals of families to the study. The Institutional Review Boards at the University of Illinois, Urbana-Champaign (#15503), the University of Houston (#0000456), California State University, Fresno (#731), and the University of Puerto Rico—Mayaguez Campus (#00002053) approved the research protocol.

### 2.4. Procedures

Upon attending a recruitment session or receiving information about the study informally, interested parents were asked to provide their contact information to research assistants or they received research staff contact information to obtain additional information. If participants met the eligibility criteria and agreed to participate in the study, they were scheduled for a focus group interview session. Before participation in the focus group interviews, parents read the consent forms in their preferred language and signed informed consent (available in English or Spanish). Trained bilingual facilitators led the focus group interviews in Spanish at convenient locations for parents and ensured privacy (e.g., conference or classrooms in community clinics, community centers, county extension offices, public schools, and university campuses). After a brief welcome, introduction, and description of focus group interview procedures (including audiotaping), parents were invited to ask questions. At the beginning of each focus group, parents completed a demographic questionnaire that included questions regarding their date and place of birth, generation status, language preference (both spoken and written), educational attainment, religion, marital status, number of children, employment, annual household income, and the number of people the income supports. Additionally, a question regarding how parents rated their general health was included. Following the demographic questionnaire, bilingual project coordinators or research assistants led each session using an interview guide that included the focus group questions and follow-up probes. A total of 13 focus groups were conducted. On average, five parents participated in each focus group session, which lasted one hour in duration. Previous studies, as well as a review of the literature, informed the development of the guide. Small focus group sessions were selected as the methodology due to the interest in having an open and in-depth discussion with parents about their physical activity practices. Parents received a 10 USD gift card or cash for their participation in this study.

### 2.5. Data Analysis

All focus group interview sessions included basic structured questions about parental views regarding PA engagement, PA attitudes, perceived PA challenges, and socio-environmental factors that might influence their own, their children, and family’s PA. All interviews were conducted in Spanish, audio-recorded, and later transcribed verbatim. Two bilingual research assistants back-translated the transcripts for accuracy purposes. Researchers followed the coding process outlined by Braun and Clarke’s six-step thematic analysis approach [26], and thematic analysis was applied to the English transcripts. Two researchers read through the transcripts multiple times and independently identified codes. Codes were then discussed and refined to further analyze the transcripts. Codes were compared throughout the process to ensure consistency and to broaden the codes when necessary. When disagreements arose, discussions took place to refine the coding schemes. After the coding process was completed, themes were selected as well as representative quotes. Researchers reviewed the quotes and agreed on their representation.

## 3. Results

### 3.1. Sample Demographic Characteristics

The sample consisted of 61 Hispanic parents (56 mothers, five fathers). As shown in Table 1, the average parent age was 41.30 years (SD = 12.29 years). Over half the parents were married (56%), had low educational attainment, and made less than 20,000 USD annually (61%). Fifty-seven percent of participants had been unemployed in the last 12 months. Of those employed, the average number of hours worked per week was 34.96 h (SD = 12.51 h). The average family household size was composed of four members (SD = 1.30). On average, parents reported having three children, having lived in the United States for almost 18 years, and speaking Spanish only (51%).

### 3.2. Focus Group Findings

The themes presented in this study represent commonalities found across the four sites. Six themes were identified, which include (1) engagement in PA as a family, (2) engagement in PA alone, (3) child engagement in PA at school, (4) exercise is for all, (5) exercise everywhere, and (6) barriers and facilitators to influence PA as a family.

#### 3.2.1. Theme 1. Physical Activity Engagement as a Family

Parents reported that they and their family members regularly participated in physical activity. Walking was the most common family PA reported by parents. Parents stated that walking was easy to incorporate into their daily family routines, such as walking kids to school and picking them up or walking as a family in the late afternoons: *“Well, I’ll tell you guys that we do it together because we’ll walk to their school, back to the house, and from the house back to the school. Whenever I take them to the park to play, I’ll take them to the slides too. And I’m chasing after them, just running around.”* Parents also state that they would walk with the entire family, or sometimes just with their spouses: *“I like to go for walks and so does my husband. We go for walks and take the kids. My kids don’t like it very much, but I force them to come.”*

Bicycling and playing soccer were other physical activities that parents reported they engaged in often as a family: *“My kids do a lot in parks like playing soccer or riding their bicycle. And well, my husband also plays a lot of soccer.”* Parents also described bicycling and playing soccer as activities that family members engaged in regularly.

Although study participants across all four sites did not mention weightlifting, we included it in this paper as it reflected the father’s influence on family PA engagement, which was alluded to in multiple focus groups. As a mother states:

*“Well, I try to do weights, but I don’t like it. I have never liked to exercise with weights, but my husband will try to include me, and he’ll show me, but I don’t like to do it. He’s started to show our daughter how to lift weights and how to use the machine, but she doesn’t enjoy it either. We include the youngest child, but it is very difficult. We’ll tell him we are going to go workout with weights, and he will join us. I’ve seen a lot of men work with weights but not women.”*

#### 3.2.2. Theme 2. Engaging in Physical Activity Alone

Some parents shared that children sometimes exercised on their own, either because they are practicing for an organized sport, they are bored, or they just enjoy doing it: *“My son likes to play soccer. So, he’ll go to the park to play or he’ll go to our backyard with the dog and he’ll practice there.”* Parents also stated that they prefer sometimes to exercise alone: *“I prefer to do stuff alone because if I take my children to the park they don’t keep up. They want to play. And when I put an aerobics video they tend to talk or laugh at me.”* Several parents stated that they preferred to exercise alone because family members were too distracting, their children did not want to join them, their job was physically demanding, and/or they exercised while the children were at school.

#### 3.2.3. Theme 3. Child Engagement in PA at School

Parents believed that their children are getting enough exercise at their schools and afterschool programs: *“And my girl, well she exercises at school, twice a week they make them run a mile. So, for them, I think that exercise is enough.”*

#### 3.2.4. Theme 4. Exercise Is for All

Regarding specific views about who should be physically active and how, parents overwhelmingly stated that everyone should exercise, that it is equally important for men, women, and children to exercise, regardless of age and body size: *“I think we all need to do exercise. It does not matter your age, if you are fat, or if you are thin, but I think exercise is for all.”* Parents did not share any preconceived notions about specifically who should exercise and instead emphasized that this should be a part of life for everyone: *“I consider that physical activity should not be limited to a certain age, because being active is healthy.”*

#### 3.2.5. Theme 5. Exercise Everywhere

Parents shared that they exercised where they can. If an area was safe and there was enough space, then they would use it: *“In my apartment in the living room. And later at the school also.”* Convenience was a major factor for parents to exercise; if a place was close, parents reported that they would be likely to use that space to exercise. Overall, parents reported that they and the family exercised most frequently at home, in their yards, at school, and parks.

#### 3.2.6. Theme 6. Barriers and Facilitators to Influence PA as a Family

Time was reported as the major deterrent to exercising, either regularly, or at all. One father shared about his wife’s lack of PA: *“[She] Doesn’t have time, she takes the children to their practices and does so with all the children.”*

One mother shared about her husband and daughter’s PA: *“My husband has wanted to go to the gym, my older daughter also, but since my husband does not have a fixed time to complete his job, he does not have time. Sometimes he does some weights here at our home.”*

Although most parents shared that they generally had safe places to exercise, some stated that there were areas that they avoided because they were not safe, in some cases, streets around their homes and local parks: *“I would like to walk, but since they robbed me at the park, well I got scared. And it was in a few parks, I tried in various parks and always something will happen. I no longer go to walk in parks. But well, the kids do exercise only at school.”*

Additionally, parents reported family sickness, children’s busy schedules, lack of motivation, and inclement weather as deterrents to being physically active. Parents also shared strategies that they would use to increase the likelihood of promoting PA within the family. For instance, mothers stated that a strategy was to involve husbands to engage their children in PA as illustrated by the following statement: *“In physical activity, well, I try to get my husband to invite our son to go play soccer even though he doesn’t like it. Well, he likes playing with him and he does a bit more of exercise.”*

Parents also stated the importance of combining exercise and a healthy diet: *“I believe there should be changes in the food and also in the exercise. Because if you are going to eat well but do not exercise, I think that will not help you. I think it has to be both.”*

## 4. Discussion

This study investigates, using a social–ecological model, Mexican and Puerto Rican parental views regarding parental factors (e.g., PA engagement and preferences, PA attitudes, perceived challenges, and facilitators) and social–environmental factors (e.g., spouse influence, places for engaging in PA) that might influence PA within the family. Unique to this study is its inclusion of an understudied sample of predominantly Spanish-speaking Mexican American and Puerto Rican mothers and a few fathers across four sites (California, Illinois, Texas, and Puerto Rico). Identifying PA perceptions is an important step in designing culturally appropriate PA interventions for underserved populations, including Mexican and Puerto Rican parents and their school-age children.

The findings of this study reveal that parents agree that everyone needs to be engaged in PA, and that it is equally important for children and adults to be active regardless of their age and body size. Parents also perceive themselves and their families as being active and engaged in multiple forms of PA. In terms of the type of PA, parents report walking as the most common PA they engage in as a family. This finding replicates previous studies, suggesting that walking is the most popular family PA among Hispanic families [17,27]. This study also suggests that bicycling and playing soccer were other common physical activities in which parents and children engaged, often as a family. Prior studies have similarly identified bicycling and playing soccer as popular physical activities among Hispanics [28,29]. Further, although parents in our study state they engage in PA as a family, parents also express their preference for exercising alone, sometimes as a way to avoid distractions. Perhaps PA interventions designed for Mexican American and Puerto Rican parents could focus on including a variety of PA, such as walking, bicycling, or playing soccer, that engage both mothers and fathers with their children as well as PA that parents can engage in alone.

In terms of perceived PA levels, study parents report that they and their children are physically active. Further, some parents perceive that their children are getting enough physical activity at school and after school programs, which is consistent with previous studies examining parental views of physical activity [15]. Parents’ optimistic view of PA engagement for themselves and family members in this study contradicts the literature reporting that Hispanic adults and children exhibit lower PA levels than other ethnic groups [30,31,32]. A large percentage of children (74%) are not meeting the recommendation of 60 min of moderate-to-vigorous physical activity per day [33]. Our results suggest the importance of providing Hispanic parents with information regarding daily PA recommendations for adults and children and PA intensity levels to ensure that parental perceptions are aligned with the recommendations. Moreover, PA recommendations for a healthy weight should be provided to Mexican and Puerto Rican parents, given the high obesity prevalence among Hispanic children [34]. The high prevalence of obesity and low PA levels found in Hispanic children are a significant public health concern. Significant concerns are due to (1) the rapid demographic growth of this population in the United States [35] and (2) the persistent trajectory of childhood obesity into adolescence and adulthood prevalent among children with obesity [36,37,38].

In this study, Hispanic parents also identify PA promoting strategies and barriers. Hispanic parents report that fathers’ involvement in PA and combining a healthy diet with exercise are valuable strategies for PA promotion among their families. Research examining the role of fathers has suggested that fathers are powerful influencers on their children’s PA, especially adolescent girls (e.g., [39]). Some research suggests that Hispanic fathers play an essential role in shaping children’s health behaviors [40,41]. Future research should consider including more fathers, as the father’s perspective is minimally represented in this study.

In addition, parents identified time constraints along with unsafe neighborhood streets and parks as major challenges to being active as a family. These findings are consistent with previous research, suggesting that lack of time and unsafe environments are the reported barriers to PA among Hispanic families [15,17,42]. Furthermore, these findings highlight the importance of providing Hispanic families with (a) PA that requires short bouts of time, which are effective in increasing PA in adults and children at home [43], and (b) tips on how to be active at home after school during weekdays and on weekends.

The study outcomes should be interpreted considering several limitations. The sample consists of mostly Spanish-speaking Mexican Americans and Puerto Ricans, limiting the generalizability of the results to another Hispanic subgroup of mothers. Additionally, although the goal of this study is to explore perceptions of parental PA views, most of the study participants were mothers with few fathers participating. However, our study reveals that, within the Hispanic family, the father can play a relevant role in modeling PA and in facilitating family interactions for PA. Thus, the inclusion of more fathers in future studies is warranted. Another limitation of this study is that, although eligibility criteria included parents having a child between the ages of 6–18, child age is not explicitly collected in this study. We recognize that this information is important to consider when examining parental perspectives regarding PA. This study did not explore other complex issues related to PA participation, such as cultural and gender differences in various PA engagement, and sibling and peer influences on PA. It would be helpful to ascertain families’ preferences for the type of PA according to child gender, the preferred location for PA, and the ideal daily time that parents are willing to devote to PA. We will explore this information in the future to incorporate new findings in curriculum programming for adaptations of the *Abriendo Caminos* curriculum [44]. Despite these limitations, this study provides valuable information regarding perceptions of Hispanic parents concerning factors that influence their PA engagement as well as their children and families. This information is critical in the development of culturally appropriate PA interventions for Hispanic families.

## Figures and Tables

**Table 1:** Parent Demographic Characteristics (N = 61).

Age M(SD)	41.30 (12.29)
Married marital status N (%)	34 (55.70)
Years of formal education M (SD)	
In United States	4.21 (5.36)
In home country	8.24 (3.79)
Employment and Income	
Weekly worked hours M (SD)	34.96 (12.51)
Unemployed N (%)	35 (57.40)
Less than 20,000 USD annually N (%)	37 (60.60)
Household size M (SD)	4.17 (1.30)
Number of children M (SD)	3.09 (1.93)
Number of years in the U.S. M (SD)	17.94 (8.30)
Language spoken N (%)	
Only Spanish	31 (50.80)
Speak Spanish better than English	19 (31.10)
Language Read N (%)	
Only Spanish	28 (45.90)
Read Spanish better than English	16 (26.20)

## Data Availability

Data are confidential because we notified the participants/IRB that the data would only be accessible to the research team. Survey data are available upon request.
